# Supplementary material for: Heparin-based hydrogel scaffolding alters the transcriptomic profile and increases the chemoresistance of MDA-MB-231 triple-negative breast cancer cells
Source: Biomater Sci. 2020 Feb 13;8(10):2786–96. doi: 10.1039/c9bm01481k (PMC7497406; doi:10.1039/c9bm01481k)
Supplement: Supplementary file 2 [file BM-008-C9BM01481K-s002.zip › Supplementary File 4/HP-BhydrogelvControl/Pathways/my_analysis.Gsea.1545201079907/HALLMARK_HYPOXIA.html]

Details for gene set HALLMARK\_HYPOXIA[GSEA]

|  || Dataset | expr.class.cls#HYD\_versus\_CONTROL.class.cls#HYD\_versus\_CONTROL\_repos |
| Phenotype | class.cls#HYD\_versus\_CONTROL\_repos |
| Upregulated in class | CONTROL |
| GeneSet | HALLMARK\_HYPOXIA |
| Enrichment Score (ES) | -0.32462752 |
| Normalized Enrichment Score (NES) | -1.5086173 |
| Nominal p-value | 0.0 |
| FDR q-value | 0.018832875 |
| FWER p-Value | 0.114 |
Table: GSEA Results Summary

  

Fig 1: Enrichment plot: HALLMARK\_HYPOXIA      
 Profile of the Running ES Score & Positions of GeneSet Members on the Rank Ordered List

  

| PROBE | DESCRIPTION (from dataset) | GENE SYMBOL | GENE\_TITLE | RANK IN GENE LIST | RANK METRIC SCORE | RUNNING ES | CORE ENRICHMENT || 1 | PHKG1 | na |  |  | 45 | 3.690 | 0.0276 | No |
| 2 | STC2 | na |  |  | 313 | 2.647 | 0.0351 | No |
| 3 | MAFF | na |  |  | 475 | 2.376 | 0.0459 | No |
| 4 | COL5A1 | na |  |  | 935 | 1.921 | 0.0374 | No |
| 5 | ENO3 | na |  |  | 1277 | 1.702 | 0.0333 | No |
| 6 | CHST2 | na |  |  | 1302 | 1.689 | 0.0458 | No |
| 7 | DUSP1 | na |  |  | 1364 | 1.645 | 0.0559 | No |
| 8 | EXT1 | na |  |  | 1463 | 1.598 | 0.0638 | No |
| 9 | PPP1R15A | na |  |  | 1561 | 1.546 | 0.0712 | No |
| 10 | ANGPTL4 | na |  |  | 1665 | 1.505 | 0.0780 | No |
| 11 | IRS2 | na |  |  | 1821 | 1.435 | 0.0816 | No |
| 12 | JUN | na |  |  | 1992 | 1.366 | 0.0837 | No |
| 13 | DDIT3 | na |  |  | 2346 | 1.225 | 0.0751 | No |
| 14 | ATF3 | na |  |  | 2583 | 1.150 | 0.0721 | No |
| 15 | SCARB1 | na |  |  | 2693 | 1.119 | 0.0754 | No |
| 16 | TPST2 | na |  |  | 3436 | 0.930 | 0.0440 | No |
| 17 | GPC1 | na |  |  | 3500 | 0.918 | 0.0481 | No |
| 18 | FOXO3 | na |  |  | 3688 | 0.873 | 0.0454 | No |
| 19 | PPFIA4 | na |  |  | 3914 | 0.830 | 0.0403 | No |
| 20 | ILVBL | na |  |  | 3917 | 0.829 | 0.0470 | No |
| 21 | B3GALT6 | na |  |  | 3941 | 0.824 | 0.0524 | No |
| 22 | NDST1 | na |  |  | 4404 | 0.732 | 0.0341 | No |
| 23 | PYGM | na |  |  | 4784 | 0.662 | 0.0196 | No |
| 24 | TPBG | na |  |  | 4814 | 0.656 | 0.0234 | No |
| 25 | NEDD4L | na |  |  | 4843 | 0.650 | 0.0272 | No |
| 26 | PRKCA | na |  |  | 4876 | 0.646 | 0.0308 | No |
| 27 | HDLBP | na |  |  | 4984 | 0.629 | 0.0303 | No |
| 28 | CHST3 | na |  |  | 5126 | 0.610 | 0.0278 | No |
| 29 | TIPARP | na |  |  | 5438 | 0.563 | 0.0160 | No |
| 30 | EGFR | na |  |  | 5500 | 0.552 | 0.0173 | No |
| 31 | PFKL | na |  |  | 5644 | 0.533 | 0.0141 | No |
| 32 | CDKN1B | na |  |  | 6043 | 0.481 | -0.0029 | No |
| 33 | FOS | na |  |  | 6167 | 0.461 | -0.0056 | No |
| 34 | JMJD6 | na |  |  | 6298 | 0.445 | -0.0088 | No |
| 35 | VHL | na |  |  | 6495 | 0.416 | -0.0157 | No |
| 36 | CTGF | na |  |  | 6894 | 0.371 | -0.0336 | No |
| 37 | TNFAIP3 | na |  |  | 6980 | 0.364 | -0.0351 | No |
| 38 | IL6 | na |  |  | 7244 | 0.333 | -0.0462 | No |
| 39 | SLC37A4 | na |  |  | 7261 | 0.331 | -0.0444 | No |
| 40 | HEXA | na |  |  | 7441 | 0.314 | -0.0512 | No |
| 41 | AMPD3 | na |  |  | 7472 | 0.311 | -0.0503 | No |
| 42 | BCL2 | na |  |  | 7623 | 0.289 | -0.0558 | No |
| 43 | FOSL2 | na |  |  | 7639 | 0.286 | -0.0543 | No |
| 44 | CDKN1C | na |  |  | 7826 | 0.269 | -0.0619 | No |
| 45 | NAGK | na |  |  | 7916 | 0.260 | -0.0644 | No |
| 46 | PPP1R3C | na |  |  | 7987 | 0.249 | -0.0661 | No |
| 47 | HOXB9 | na |  |  | 8151 | 0.240 | -0.0727 | No |
| 48 | PIM1 | na |  |  | 8219 | 0.229 | -0.0744 | No |
| 49 | SLC6A6 | na |  |  | 8243 | 0.227 | -0.0737 | No |
| 50 | CP | na |  |  | 8317 | 0.218 | -0.0758 | No |
| 51 | SDC3 | na |  |  | 8522 | 0.202 | -0.0849 | No |
| 52 | ADORA2B | na |  |  | 8611 | 0.191 | -0.0879 | No |
| 53 | NDST2 | na |  |  | 8759 | 0.171 | -0.0943 | No |
| 54 | PFKFB3 | na |  |  | 8864 | 0.157 | -0.0985 | No |
| 55 | STBD1 | na |  |  | 9033 | 0.149 | -0.1061 | No |
| 56 | F3 | na |  |  | 9090 | 0.142 | -0.1079 | No |
| 57 | KDM3A | na |  |  | 9405 | 0.105 | -0.1235 | No |
| 58 | ADM | na |  |  | 9646 | 0.081 | -0.1355 | No |
| 59 | ANKZF1 | na |  |  | 9819 | 0.062 | -0.1440 | No |
| 60 | BCAN | na |  |  | 9870 | 0.056 | -0.1462 | No |
| 61 | PGF | na |  |  | 10123 | 0.025 | -0.1592 | No |
| 62 | GAA | na |  |  | 10143 | 0.023 | -0.1600 | No |
| 63 | GCNT2 | na |  |  | 10333 | 0.014 | -0.1698 | No |
| 64 | MAP3K1 | na |  |  | 10425 | 0.004 | -0.1746 | No |
| 65 | DTNA | na |  |  | 10478 | -0.002 | -0.1773 | No |
| 66 | GRHPR | na |  |  | 10557 | -0.011 | -0.1813 | No |
| 67 | ZFP36 | na |  |  | 10619 | -0.019 | -0.1843 | No |
| 68 | GYS1 | na |  |  | 10792 | -0.037 | -0.1931 | No |
| 69 | P4HA2 | na |  |  | 10841 | -0.045 | -0.1952 | No |
| 70 | PDK1 | na |  |  | 10844 | -0.045 | -0.1950 | No |
| 71 | ENO2 | na |  |  | 11078 | -0.075 | -0.2066 | No |
| 72 | NR3C1 | na |  |  | 11080 | -0.075 | -0.2061 | No |
| 73 | IGFBP1 | na |  |  | 11345 | -0.095 | -0.2191 | No |
| 74 | GPI | na |  |  | 11431 | -0.105 | -0.2228 | No |
| 75 | PFKP | na |  |  | 11433 | -0.105 | -0.2220 | No |
| 76 | PLAUR | na |  |  | 11509 | -0.112 | -0.2250 | No |
| 77 | TGFB3 | na |  |  | 11572 | -0.122 | -0.2272 | No |
| 78 | SIAH2 | na |  |  | 11609 | -0.127 | -0.2281 | No |
| 79 | CDKN1A | na |  |  | 11746 | -0.145 | -0.2341 | No |
| 80 | SELENBP1 | na |  |  | 11758 | -0.146 | -0.2335 | No |
| 81 | MT1E | na |  |  | 11922 | -0.166 | -0.2407 | No |
| 82 | ETS1 | na |  |  | 12310 | -0.215 | -0.2593 | No |
| 83 | KLF6 | na |  |  | 12855 | -0.265 | -0.2857 | No |
| 84 | SLC25A1 | na |  |  | 13006 | -0.284 | -0.2913 | No |
| 85 | EFNA1 | na |  |  | 13011 | -0.285 | -0.2891 | No |
| 86 | AKAP12 | na |  |  | 13048 | -0.290 | -0.2887 | No |
| 87 | XPNPEP1 | na |  |  | 13136 | -0.304 | -0.2908 | No |
| 88 | SLC2A5 | na |  |  | 13246 | -0.316 | -0.2939 | No |
| 89 | ZNF292 | na |  |  | 13298 | -0.325 | -0.2940 | No |
| 90 | FBP1 | na |  |  | 13369 | -0.333 | -0.2950 | No |
| 91 | PDGFB | na |  |  | 13460 | -0.347 | -0.2969 | No |
| 92 | SDC4 | na |  |  | 13470 | -0.349 | -0.2945 | No |
| 93 | KLF7 | na |  |  | 13490 | -0.352 | -0.2926 | No |
| 94 | BHLHE40 | na |  |  | 13518 | -0.356 | -0.2912 | No |
| 95 | PGM2 | na |  |  | 13527 | -0.357 | -0.2887 | No |
| 96 | CSRP2 | na |  |  | 13680 | -0.382 | -0.2936 | No |
| 97 | ISG20 | na |  |  | 13723 | -0.391 | -0.2926 | No |
| 98 | HSPA5 | na |  |  | 13730 | -0.393 | -0.2897 | No |
| 99 | TGM2 | na |  |  | 13799 | -0.403 | -0.2900 | No |
| 100 | GALK1 | na |  |  | 14078 | -0.428 | -0.3012 | No |
| 101 | INHA | na |  |  | 14275 | -0.458 | -0.3077 | No |
| 102 | CCNG2 | na |  |  | 14302 | -0.464 | -0.3053 | No |
| 103 | DDIT4 | na |  |  | 14408 | -0.478 | -0.3070 | No |
| 104 | CITED2 | na |  |  | 14498 | -0.490 | -0.3077 | No |
| 105 | PNRC1 | na |  |  | 14528 | -0.496 | -0.3052 | No |
| 106 | EFNA3 | na |  |  | 14572 | -0.502 | -0.3034 | No |
| 107 | WSB1 | na |  |  | 14978 | -0.558 | -0.3201 | Yes |
| 108 | HK1 | na |  |  | 15003 | -0.562 | -0.3168 | Yes |
| 109 | WISP2 | na |  |  | 15043 | -0.569 | -0.3142 | Yes |
| 110 | SDC2 | na |  |  | 15052 | -0.571 | -0.3100 | Yes |
| 111 | SLC2A1 | na |  |  | 15114 | -0.583 | -0.3085 | Yes |
| 112 | SAP30 | na |  |  | 15285 | -0.614 | -0.3124 | Yes |
| 113 | ALDOA | na |  |  | 15303 | -0.618 | -0.3083 | Yes |
| 114 | HMOX1 | na |  |  | 15431 | -0.638 | -0.3098 | Yes |
| 115 | TPI1 | na |  |  | 15532 | -0.658 | -0.3097 | Yes |
| 116 | HK2 | na |  |  | 15767 | -0.695 | -0.3163 | Yes |
| 117 | BTG1 | na |  |  | 15833 | -0.711 | -0.3140 | Yes |
| 118 | HS3ST1 | na |  |  | 16034 | -0.755 | -0.3184 | Yes |
| 119 | CA12 | na |  |  | 16047 | -0.759 | -0.3128 | Yes |
| 120 | PLAC8 | na |  |  | 16134 | -0.774 | -0.3110 | Yes |
| 121 | IGFBP3 | na |  |  | 16184 | -0.784 | -0.3073 | Yes |
| 122 | KLHL24 | na |  |  | 16186 | -0.785 | -0.3009 | Yes |
| 123 | STC1 | na |  |  | 16195 | -0.787 | -0.2950 | Yes |
| 124 | NFIL3 | na |  |  | 16230 | -0.795 | -0.2903 | Yes |
| 125 | EDN2 | na |  |  | 16304 | -0.811 | -0.2875 | Yes |
| 126 | CAV1 | na |  |  | 16368 | -0.825 | -0.2841 | Yes |
| 127 | P4HA1 | na |  |  | 16447 | -0.842 | -0.2814 | Yes |
| 128 | PGM1 | na |  |  | 16509 | -0.856 | -0.2777 | Yes |
| 129 | PPARGC1A | na |  |  | 16612 | -0.876 | -0.2759 | Yes |
| 130 | TGFBI | na |  |  | 16797 | -0.927 | -0.2780 | Yes |
| 131 | SLC2A3 | na |  |  | 16869 | -0.946 | -0.2741 | Yes |
| 132 | MT2A | na |  |  | 17171 | -1.038 | -0.2815 | Yes |
| 133 | ATP7A | na |  |  | 17248 | -1.066 | -0.2768 | Yes |
| 134 | PRDX5 | na |  |  | 17276 | -1.076 | -0.2695 | Yes |
| 135 | RRAGD | na |  |  | 17307 | -1.086 | -0.2622 | Yes |
| 136 | PAM | na |  |  | 17315 | -1.089 | -0.2537 | Yes |
| 137 | UGP2 | na |  |  | 17321 | -1.092 | -0.2451 | Yes |
| 138 | LOX | na |  |  | 17344 | -1.100 | -0.2374 | Yes |
| 139 | PLIN2 | na |  |  | 17478 | -1.144 | -0.2351 | Yes |
| 140 | VLDLR | na |  |  | 17499 | -1.153 | -0.2268 | Yes |
| 141 | PDK3 | na |  |  | 17601 | -1.198 | -0.2223 | Yes |
| 142 | NDRG1 | na |  |  | 17623 | -1.208 | -0.2136 | Yes |
| 143 | RBPJ | na |  |  | 17645 | -1.218 | -0.2048 | Yes |
| 144 | TPD52 | na |  |  | 17682 | -1.234 | -0.1967 | Yes |
| 145 | TES | na |  |  | 17844 | -1.305 | -0.1946 | Yes |
| 146 | IDS | na |  |  | 17889 | -1.328 | -0.1861 | Yes |
| 147 | ALDOC | na |  |  | 18115 | -1.452 | -0.1861 | Yes |
| 148 | RORA | na |  |  | 18188 | -1.490 | -0.1778 | Yes |
| 149 | AK4 | na |  |  | 18295 | -1.561 | -0.1707 | Yes |
| 150 | KDELR3 | na |  |  | 18309 | -1.571 | -0.1586 | Yes |
| 151 | ERRFI1 | na |  |  | 18341 | -1.595 | -0.1473 | Yes |
| 152 | CASP6 | na |  |  | 18493 | -1.701 | -0.1414 | Yes |
| 153 | GLRX | na |  |  | 18498 | -1.703 | -0.1278 | Yes |
| 154 | SRPX | na |  |  | 18537 | -1.735 | -0.1157 | Yes |
| 155 | MXI1 | na |  |  | 18597 | -1.777 | -0.1043 | Yes |
| 156 | GBE1 | na |  |  | 18637 | -1.827 | -0.0915 | Yes |
| 157 | BNIP3L | na |  |  | 18688 | -1.884 | -0.0789 | Yes |
| 158 | FAM162A | na |  |  | 18694 | -1.889 | -0.0638 | Yes |
| 159 | TMEM45A | na |  |  | 18709 | -1.902 | -0.0491 | Yes |
| 160 | CXCR4 | na |  |  | 18827 | -2.050 | -0.0386 | Yes |
| 161 | PGK1 | na |  |  | 18894 | -2.154 | -0.0245 | Yes |
| 162 | LXN | na |  |  | 18938 | -2.243 | -0.0086 | Yes |
| 163 | S100A4 | na |  |  | 19100 | -2.731 | 0.0051 | Yes |
Table: GSEA details [plain text format]

  

Fig 2: HALLMARK\_HYPOXIA      
 Blue-Pink O' Gram in the Space of the Analyzed GeneSet

  

Fig 3: HALLMARK\_HYPOXIA: Random ES distribution      
 Gene set null distribution of ES for **HALLMARK\_HYPOXIA**
